# Back to Basics in Paediatric Pneumonia—Defining a Breath and Setting Reference Standards to Innovate Respiratory Rate Counting

**DOI:** 10.1093/tropej/fmaa112

**Published:** 2020-12-05

**Authors:** King Carina, Baker Kevin, Nantanda Rebecca, Quique Bassat, Qazi Shamim Ahmad, McCollum Eric D.

**Affiliations:** 1 Department of Global Public Health, Karolinska Institutet, Sweden; 2 Institute for Global Health, University College London, UK; 3 Malaria Consortium, UK; 4 Makerere University Lung Institute, Makerere University College of Health Sciences, Uganda; 5 ISGlobal, Hospital Clínic – Universitat de Barcelona, Barcelona, Spain; 6 Centro de Investigação em Saúde de Manhiça (CISM), Maputo, Mozambique; 7 ICREA, Pg. Lluís Companys 23, Barcelona 08010, Spain; 8 Pediatrics Department, Hospital Sant Joan de Déu, Universitat de Barcelona, Esplugues, Barcelona, Spain; 9 Consorcio de Investigación Biomédica en Red de Epidemiología y Salud Pública (CIBERESP), Madrid, Spain; 10 Consultant (Retired World Health Organisation Staff), Geneva, Switzerland; 11 Global Program in Respiratory Sciences, Eudowood Division of Pediatric Respiratory Sciences, Department of Pediatrics, Johns Hopkins School of Medicine, USA; 12 Department of International Health, Johns Hopkins Bloomberg School of Public Health, USA

Every November 12th, the global health community hosts World Pneumonia Day, and in 2020, on the 11th iteration, we find ourselves once more facing the fact that pneumonia is the leading global cause of infectious mortality amongst children under-5 years old. Many regions are not within sight of the Sustainable Development Goal 3.2 target. And yet, this situation may be less surprising given we are—11 years later—still discussing the basic question of how we define (and count) a child’s single breath.

The cornerstone of paediatric pneumonia diagnosis in most low- and middle-income country (LMIC) settings, and particularly in primary care, is the evaluation of respiratory rate (RR), as part of the clinical algorithms that constitute the Integrated Management of Childhood Illness (IMCI). The introduction of this standardized syndromic approach through the WHO case management guidelines has resulted in significant pneumonia mortality reductions [1]. The current standard of determining RR is visually counting the number of breaths a child takes over an uninterrupted 60-second period, using a stopwatch or RR counting timer. However, this can be unreliable (especially amongst younger and/or uncooperative children), challenging in time-limited high-volume settings and often not routinely done by healthcare workers. Additionally, timers are often unavailable [2–4]. Therefore, the lack of low-cost, accurate and easy-to-use RR tools limits diagnostic quality of care, in turn affecting treatment decision-making and health outcomes.

Alternative RR counting tools for LMIC settings have been proposed (e.g. counting beads) and new technologies are coming to market harnessing mHealth, accelerometers and photoplethysmography. Major barriers to adoption include determining real-world performance, lack of consensus on suitable reference standards and benchmarking against current standard of care [5, 6]. In fact, there is no clear description of a ‘breath’ within WHO training tools or guidelines. While a ‘breath’ may seem so intuitive to not warrant defining, this is not necessarily true when comparing novel technologies. A breath could be defined a number of ways, including the observation of one full inhalation and exhalation, a measurable chest movement, blood volume fluctuations, a measurement of ventilation or gas exchange (capnography) [5]. It is unsurprising that these could lead to different RR counts and be the difference between a pneumonia diagnosis or not.

To move forward, expert consensus on defining a breath is fundamental. It is important that such a definition retains pragmatism and generalizability across healthcare settings, realistically meaning manual breath counts through non-invasive observation in a consistent way. While this is inherently subjective, we can take lessons from other diagnostics which also suffer from observer variability. For these, expert adjudication panels have been central. The WHO methodology for interpreting chest radiographs for vaccine studies includes objective, measurable criteria applied by trained readers to achieve interpretation, reproducibility and generalizability. We acknowledge that this is not perfect, but provides sufficient standardization to inform landmark studies [7–9]. These methods have successfully been adapted and applied to evaluate digital lung auscultation technology on children in LMICs [10].

Secondly, we see the need for an open-access repository of annotated videos of children breathing for at least 60 seconds alongside the protocol for assigning RR, to act as a quality-controlled reference standard. This would support the training of healthcare providers in measuring RR and be a resource for the development and/or validation of new RR technologies. The repository must include a fair number of infants <2 months of age, in whom there is more RR variation, and ensure sufficient diversity in key demographic factors and clinical states.

An exciting opportunity with establishing a video library is the ability to apply artificial intelligence. Artificial intelligence applications within healthcare have evolved significantly, with several examples of decision-support tools designed to aid diagnosis and prognosis. This is especially the case with image-based machine learning methods which have reached expert-level accuracy in several tasks [11], and show promise in the interpretation of video data for vital sign monitoring. Indeed, RR has already been successfully extracted from video data [12]. However, models to date have not explicitly focussed on children or those with pneumonia. It is crucial that novel RR tools be tested and validated in the context in which they will be used, i.e. on children, across the range of disease severity and healthcare settings. The behaviour and illness profiles of children in outpatient clinics can differ greatly from children in hospitals, and these differences may influence technological performance. Therefore, tools designed mainly for outpatient use should be scrutinized in this setting, and similarly, devices intended for hospital use should be tested in that context. It would be a strength for reference videos to reflect this diversity of context.

The COVID-19 pandemic has shone a bright and unforgiving light on one of the major barriers to reducing pneumonia mortality—the lack of a sustainable oxygen ecosystem to meet clinical demands. A positive legacy of this pandemic should be the ability to better treat severe pneumonia cases. However, the first step in achieving effective treatment is accurate diagnosis, and therefore, we need to first remember the basics of a breath.
